# Functional connectivity and microstructural white matter changes in phenocopy frontotemporal dementia

**DOI:** 10.1007/s00330-016-4490-4

**Published:** 2016-07-19

**Authors:** R. Meijboom, R. M. E. Steketee, I. de Koning, R. J. Osse, L. C. Jiskoot, F. J. de Jong, A. van der Lugt, J. C. van Swieten, M. Smits

**Affiliations:** 1 Radiology and Nuclear Medicine, Erasmus MC – University Medical Centre, Rotterdam, The Netherlands; 2 Neuropsychology, Erasmus MC – University Medical Centre, Rotterdam, The Netherlands; 3 Psychiatry, Erasmus MC – University Medical Centre, Rotterdam, The Netherlands; 4 Neurology, Erasmus MC – University Medical Centre, Rotterdam, The Netherlands

**Keywords:** Frontotemporal dementia, Diffusion tensor imaging, Functional magnetic resonance imaging, White matter, Diagnosis

## Abstract

**Objectives:**

Phenocopy frontotemporal dementia (phFTD) is a rare and poorly understood clinical syndrome. PhFTD shows core behavioural variant FTD (bvFTD) symptoms without associated cognitive deficits and brain abnormalities on conventional MRI and without progression. In contrast to phFTD, functional connectivity and white matter (WM) microstructural abnormalities have been observed in bvFTD. We hypothesise that phFTD belongs to the same disease spectrum as bvFTD and investigated whether functional connectivity and microstructural WM changes similar to bvFTD are present in phFTD.

**Methods:**

Seven phFTD patients without progression or alternative psychiatric diagnosis, 12 bvFTD patients and 17 controls underwent resting state functional MRI (rs-fMRI) and diffusion tensor imaging (DTI). Default mode network (DMN) connectivity and WM measures were compared between groups.

**Results:**

PhFTD showed subtly increased DMN connectivity and subtle microstructural changes in frontal WM tracts. BvFTD showed abnormalities in similar regions as phFTD, but had lower increased DMN connectivity and more extensive microstructural WM changes.

**Conclusions:**

Our findings can be interpreted as neuropathological changes in phFTD and are in support of the hypothesis that phFTD and bvFTD may belong to the same disease spectrum. Advanced MRI techniques, objectively identifying brain abnormalities, would therefore be potentially suited to improve the diagnosis of phFTD.

***Key points*:**

• *PhFTD shows brain abnormalities that are similar to bvFTD.*

• *PhFTD shows increased functional connectivity in the parietal default mode network.*

• *PhFTD shows microstructural white matter abnormalities in the frontal lobe.*

• *We hypothesise phFTD and bvFTD may belong to the same disease spectrum.*

**Electronic supplementary material:**

The online version of this article (doi:10.1007/s00330-016-4490-4) contains supplementary material, which is available to authorized users.

## Introduction

Phenocopy (or nonprogressive) frontotemporal dementia (phFTD) is a rare and poorly understood syndrome, which was only recently described by Davies et al. 2006 [1] in a subgroup of behavioural variant FTD (bvFTD) patients who had a better prognosis than expected.

PhFTD symptomatology is very similar to that of bvFTD, but some aspects of phFTD are essentially different. In phFTD, core bvFTD symptoms, such as apathy, behavioural disinhibition and loss of insight [2], are generally not accompanied by cognitive and brain abnormalities as is the case in bvFTD. PhFTD patients show a cognitive profile that ranges from normal to suggesting FTD [3–6] and have a relatively intact performance of daily living activities (ADL) [2, 6]. These clinical features in phFTD appear stable over time, whereas in bvFTD patients rapid progression of cognitive deficits is evident [1, 6–8]. On conventional (structural) magnetic resonance imaging (MRI), phFTD patients show no or only borderline abnormalities [1, 8] in the frontal and temporal regions, which are typically affected in bvFTD [9]. Positron emission tomography (PET) does not show the frontal hypometabolism that is observed in bvFTD [10]. As bvFTD patients may initially also present without structural MRI abnormalities, early stage distinction between phFTD and bvFTD may be difficult.

A pathophysiological explanation for phFTD symptomatology is currently unavailable. Patients often remain undiagnosed or receive an alternative psychiatric diagnosis. Additionally, they are occasionally found to be C9orf72 mutation carriers [11]. It is therefore of importance to investigate the presence of possible brain abnormalities underlying their symptoms using more advanced MRI techniques such as resting state functional MRI (rs-fMRI) and diffusion tensor imaging (DTI). These techniques measure subtle brain changes so far left unexplored by looking at functional connectivity and microstructural white matter (WM). A well-defined network showing functional abnormalities in bvFTD is the default mode network (DMN). In bvFTD, parietal regions of the DMN show increased connectivity [12, 13]. Frontal DMN connectivity changes are more ambiguous, found to be either increased [14] or decreased [12]. Such functional changes are thought to precede grey matter atrophy appearing at the later stages of bvFTD [12, 15]. WM abnormalities in bvFTD are found mainly in frontal and temporal areas such as the uncinate fasciculus (UF), cingulum and genu of the corpus callosum (CC) [16]. Similar regions are found to be already affected in asymptomatic FTD mutation carriers [15].

As phFTD patients present with behavioural symptoms similar to bvFTD, we hypothesise that phFTD and bvFTD may belong to the same disease spectrum. This study investigates whether phFTD patients have underlying brain abnormalities that are similar to those seen in bvFTD patients: functional brain abnormalities expressed as DMN connectivity changes and microstructural WM abnormalities expressed as diffusion changes.

## Methods

### Participants

All patients were recruited in the Alzheimer Centre Southwest Netherlands. PhFTD patients (aged 40-75 years) with prominent behavioural changes interfering with social functioning, consisting of disinhibition and/or apathy and/or stereotypy; no reported progression 1 year after initial routine diagnostic workup, and bvFTD patients (aged 45-70 years) with a diagnosis of bvFTD [17]; a clinical dementia rating scale score of ≤1; and a Mini-Mental State Examination [18] (MMSE) score of ≥ 20 were included in the study.

Patients with other neurological disorders, past or current substance abuse or other psychiatric diagnosis were excluded. PhFTD patients with a diagnosis of dementia and missing heteroanamnesis and bvFTD patients with a different cause of dementia were also excluded.

Healthy controls (aged 60-70 years), without neurological or psychiatric history, were recruited through advertisement. They were matched for gender with phFTD patients and for age with all patients.

The study was approved by the local medical ethics committee. All participants gave written informed consent.

### Psychiatric, neuropsychological and genetic mutation assessment

PhFTD patients underwent full psychiatric assessment as part of this study to exclude alternative diagnoses. Additionally, their DNA was tested for the C9orf72 mutation.

All participants underwent a full neuropsychological assessment and an MMSE [18]. Six phFTD patients had a cognitive profile suggestive of FTD, but showed no progression relative to previous neuropsychological testing consistent with the phFTD criteria. One phFTD patient had a normal cognitive profile. Mean interval between current and first neuropsychological testing was 36 months (range: 8-71 months). The cognitive profile was consistent with bvFTD for bvFTD patients and normal for controls.

### Image acquisition

Scanning was performed on two 3T GE Discovery MR750 systems (GE Healthcare, Milwaukee, WI, US) with identical protocols. PhFTD patients and 12 controls were scanned on one and bvFTD patients and 8 controls on the other scanner. All participants underwent a high-resolution three-dimensional (3D) inversion recovery (IR) fast spoiled gradient echo (FSPGR) T1-weighted image for anatomical reference and a functional gradient echo echo planar imaging (EPI) and DTI spin echo EPI with full coverage of the supratentorial brain (Table 1). During the functional scan participants were instructed to focus on a fixation cross, to think of nothing in particular and to stay awake.

**Table 1:** Acquisition parameters

	T1w	fMRI	DTI
FOV (mm)	240	240	240
TE (ms)	3.06	30	84.2*
TR (ms)	7.90	3000	7925
ASSET factor	2	2	2
Flip angle	120°	90°	90°
Acquisition matrix	240 × 240	96 × 96	128 × 128
Slice thickness (mm)	1	3	2.5
Volumes (slices per volume)	1 (176)	200 (44)	28 (59)
Duration (min)	4.41	10.00	3.50
Diffusion-weighted directions	n/a	n/a	25
Maximum b-value (s/mm^2^)	n/a	n/a	1000
TI (ms)	450	n/a	n/a

T1w = T1-weighted, fMRI = functional magnetic resonance imaging, DTI = diffusion tensor imaging, FOV = field of view, TE = echo time, TR = repetition time, ASSET = array spatial sensitivity encoding technique, TI = inversion time

*TE for DTI was set to the minimum. This number represents the average TE. The range of TE was 81.9-90.8 ms

### Structural MRI analysis

Whole-brain grey matter (GM) volume was calculated for each participant according to the methods described in Bron et al. 2014 [19]. For each participant, whole-brain GM volume was divided by their individual intracranial volume (ICV) to correct for head size (expressed as % ICV) [20–22] and referred to as normalised GM (nGM). These values (%ICV) were then averaged per group, resulting in a mean nGM volume expressed as %ICV. These group means were then compared between groups using a one-way ANOVA and post-hoc Bonferroni tests (SPSS21, USA).

### Rs-fMRI analysis

Rs-fMRI data were analysed using FMRIB Software library (FSL4.1.9, UK) (see supplement section 1 for more detail). The brain was extracted using the Brain Extraction Tool (BET) [23]. Then, MELODIC independent component analysis was used for pre-processing of the functional data and establishing the DMN component. Due to the small sample size, only one network of interest could be investigated. The DMN was chosen as it is a well-defined functional network showing functional abnormalities in bvFTD, allowing for meaningful comparison between phFTD and bvFTD. Hereafter, the participant-specific DMN was identified using dual regression [24]. Subsequently, Randomize [25] was used to assess between-group DMN connectivity differences using a one-way ANCOVA with three groups (phFTD≠bvFTD≠controls) and GM volume as covariate. Main effects were investigated and six t-contrasts (phFTD>HC, HC>phFTD, bvFTD>HC, HC>bvFTD, phFTD>bvFTD, bvFTD>phFTD) were constructed to assess post-hoc between-group differences.

Cluster was used to extract cluster information. Results [*p*_corrected_ < 0.05, cluster size (k) ≥ 1; p_uncorrected_ <0.05, k ≥ 20] were visualised using FSLview. Structural atlases implemented in FSLView were used to anatomically identify the DMN regions.

### DTI analysis

Data were corrected for motion, eddy currents and EPI distortions using ExploreDTI [26]. Further analyses were performed with FSL (5.0.2.2, UK) (see supplement section 1 for more detail). BET [23] was used to create skull-stripped binary masks, after which DTIFIT [27] was used to reconstruct diffusion tensors and to create subject images for all WM measures, i.e. fractional anisotropy (FA), mean diffusivity (MD), axial diffusivity (AxD) and radial diffusivity (RD). Then, registration was performed using Tract-Based Spatial Statistics (TBSS) [28], resulting in subject-specific WM skeletons for each WM measure. Subsequently, Randomize [25] was used to assess between-group WM measure differences using a one-way ANOVA with the same groups and post-hoc t-tests as in the rs-fMRI analysis.

Cluster was used to extract cluster information. Results (*p*_corrected_ < 0.05, k ≥ 20) were visualised using FSLView. The WM atlases implemented in FSLView were used to anatomically identify the WM regions.

## Results

### Participant and disease characteristics

Data of 7 phFTD patients, 12 bvFTD patients (9 for rs-fMRI analysis) and 17 controls were used for the current analyses (Table 2). In total, 9 phFTD patients (all male), 12 bvFTD patients (7 male) and 20 healthy controls (all male) were originally included in the study. Two phFTD patients and three controls were excluded from the analysis (see supplement section 2). Three bvFTD patients had missing rs-fMRI data. One phFTD patient’s follow-up was just under 1 year (11 months), for logistical reasons. The clinical diagnosis phFTD was established based on the clinical profile and lack of disease progression.

**Table 2:** Demographic characteristics

Group	*N*	Mean age (years)	Mean MMSE
PhFTD	7 (all male)	63.4 (4.8)	26.6 (1.4)
BvFTD	12 (7 male)	60.2 (7.6)	26.6 (2.8)
Controls	17 (all male)	64.1 (3.3)	28.2 (1.5)

PhFTD = phenocopy FTD, BvFTD = behavioural FTD, N = sample size, values given as mean (SD), MMSE = Mini-mental State Examination

Age [H (2) = 2,23 *p* > 0.05, Table 2] and the MMSE score did not differ between groups [H (2) = 5,93, *p* > 0.05, Table 2].

None of the phFTD patients received an alternative psychiatric diagnosis that could explain their behavioural symptoms. Additionally, none carried the C9orf72 mutation.

### Structural MRI

The three groups showed a difference in nGM [H (2) = 16.38, *p* < 0.05], with a mean of 0.32 %ICV (SD 0.02) for phFTD, 0.29 %ICV (SD 0.04) for bvFTD and 0.35 %ICV (SD 0.02) for controls. Compared with controls, both phFTD patients (*p* = 0.013) and bvFTD patients (*p* < 0.001) had lower nGM volume. nGM volume was not different between phFTD and bvFTD patients (*p* = 0.359).

### Functional connectivity

**Fig. 1:**
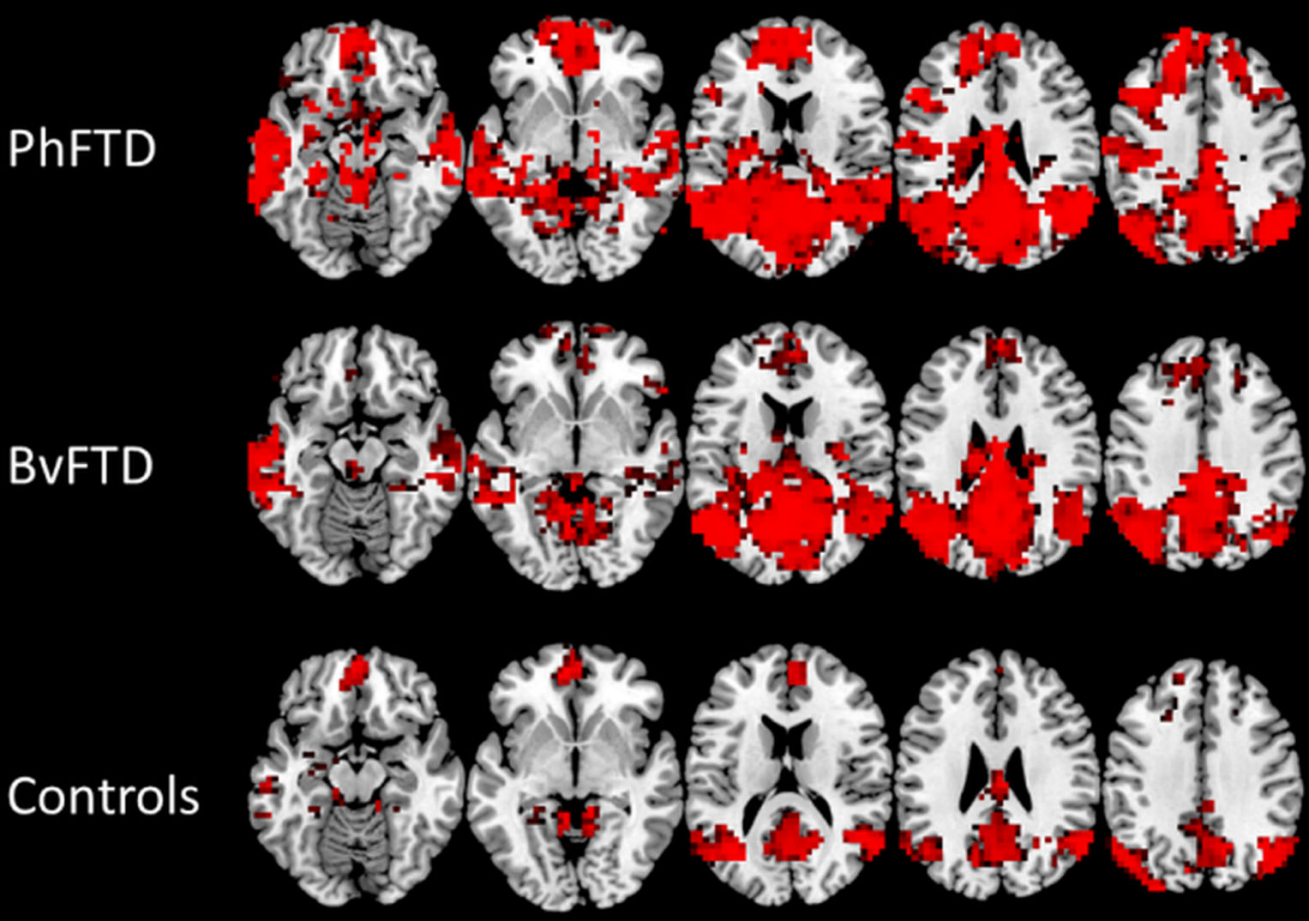
Mean default mode network (DMN) connectivity [*p* < 0.05, not corrected for multiple comparisons, but Bonferroni corrected (*p* < 0.05) for multiple contrasts; k≥20] in phenocopy frontotemporal dementia (phFTD), behavioural variant FTD (bvFTD) and controls

PhFTD and bvFTD patients (Fig. 1, supplement Table 1A) showed connectivity in all regions of the DMN. Controls showed connectivity in all DMN regions except the right lateral temporal cortex (LTC) (Fig. 1, supplement Table 1A).

**Fig. 2:**
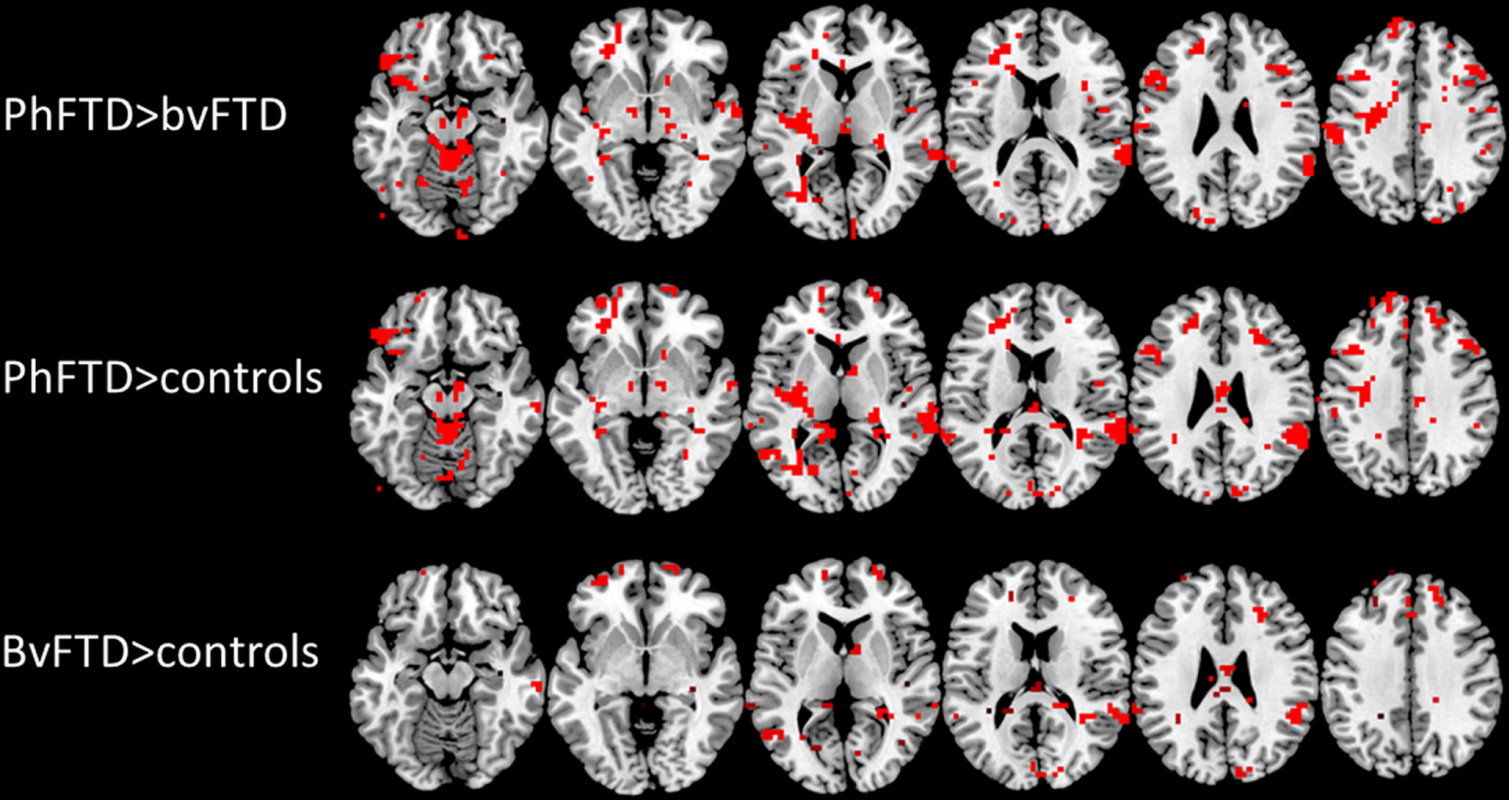
Post-hoc t-test comparisons (phFTD>bvFTD, phFTD>controls, bvFTD>controls) showing between-group DMN connectivity differences (*p* < 0.05, not corrected for multiple comparisons, but within the constraints of the omnibus f-test (*p* < 0.05, Bonferroni corrected for multiple contrasts); k ≥ 20).DMN = default mode network, phFTD = phenocopy frontotemporal dementia, bvFTD = behavioural variant FTD

PhFTD patients compared with controls showed increased DMN connectivity in the bilateral medial prefrontal cortex (mPFC), LTC and inferior parietal lobule (IPL) and in the left posterior cingulate cortex (PCC)/precuneus (Fig. 2, supplement Table 1B).

BvFTD patients compared with controls showed increased DMN connectivity in the bilateral mPFC and right LTC and IPL (Fig. 2, supplement Table 1B) and decreased DMN connectivity in the more posterior right IPL.

PhFTD patients compared with bvFTD patients showed increased DMN connectivity in the bilateral mPFC and LTC and right IPL (Fig. 2, supplement Table 1B).

### Microstructural WM

**Fig. 3:**
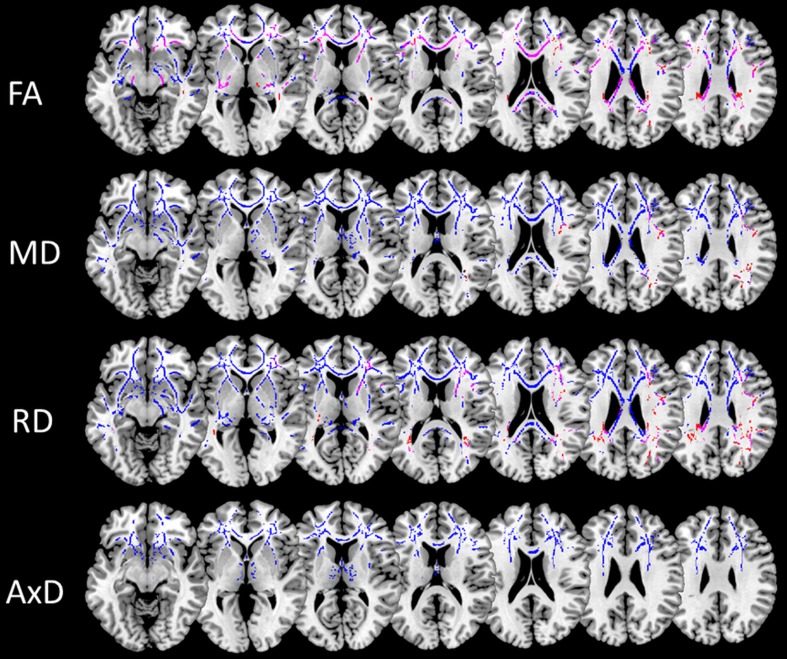
Post-hoc t-test of microstructural white matter changes for phFTD (*p*_corrected_ < 0.05; k ≥ 20) and bvFTD (*p*_corrected_ < 0.05; k ≥ 20) in comparison with controls. Lower FA and higher MD, RD and AxD in comparison with controls are shown in phFTD in red and in bvFTD in blue. WM regions showing overlapping abnormalities in phFTD and bvFTD are shown in pink. PhFTD = phenocopy frontotemporal dementia, bvFTD = behavioural variant frontotemporal dementia, FA = fractional anisotropy, MD = mean diffusivity, RD = radial diffusivity, AxD = axial diffusivity

PhFTD and bvFTD patients compared with controls showed decreased FA and increased RD and MD mainly in the frontal and temporoparietal WM (Fig. 3, supplement Tables 2A-C), such as the cingulum (both cingulate and hippocampus portion), inferior fronto-occipital fasciculus (IFOF), superior longitudinal fasciculus (SLF), corpus callosum and uncinate fasciculus. In bvFTD patients in comparison with controls increased AxD was observed in these regions as well (Fig. 3, supplement Table 2D).

**Fig. 4:**
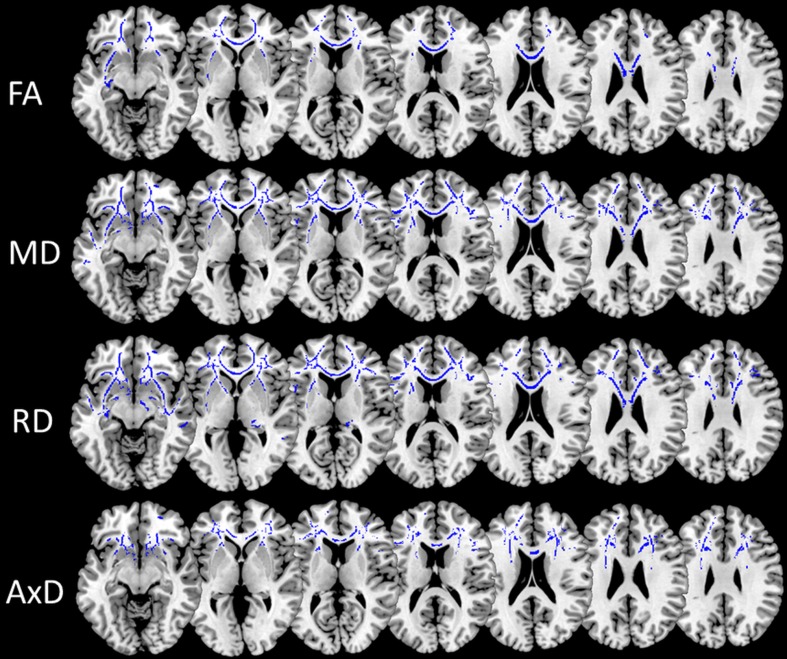
Post-hoc t-test microstructural white matter changes for bvFTD in comparison with phFTD (*p*_corrected_ < 0.05; k ≥ 20). Lower FA and higher MD, RD and AxD in bvFTD are shown in blue. PhFTD = phenocopy frontotemporal dementia, bvFTD = behavioural variant FTD, FA = fractional anisotropy, MD = mean diffusivity, RD = radial diffusivity, AxD = axial diffusivity

BvFTD patients compared with phFTD patients showed decreased FA and increased AxD in frontal WM and increased MD and RD in frontotemporal WM, mainly in the cingulum (cingulate portion), IFOF, SLF, corpus callosum and uncinate fasciculus (Fig. 4, supplement Tables 2A-D).

## Discussion

To the best of our knowledge, this is the first study demonstrating functional connectivity changes and microstructural WM abnormalities in phFTD. There was increased DMN connectivity in nearly all regions of the DMN and abnormal microstructural WM in the frontal and temporoparietal lobes. These changes were similar to the changes observed in bvFTD, supporting our hypothesis that phFTD may belong to the same disease spectrum as bvFTD. Specifically, bvFTD also showed higher connectivity in DMN regions, but to a lesser extent than in phFTD, and microstructural WM abnormalities in the frontal and temporoparietal lobes, but more pronounced than in phFTD.

DMN connectivity was increased, albeit to a moderate extent, in both phFTD and bvFTD. As there are overt behavioural symptoms in phFTD, as well as frequent neuropsychological abnormalities, it is not surprising to observe functional brain abnormalities. Increased functional connectivity reflects changes in neuronal activity becoming more congruent between regions. This may point to a brain mechanism compensating for early diminished neuronal functioning [29, 30]. The degree of increased connectivity may reflect the brain’s remaining ability of compensation, and in case of advanced neuronal dysfunctioning, this ability may no longer present, resulting in decreased connectivity. This theory may explain why we observe higher connectivity in phFTD than in bvFTD. As pronounced cortical atrophy is evident in bvFTD but not in phFTD [1, 8, 9, 31], neuronal dysfunctioning is likely much more prominent in bvFTD. This means that more relatively preserved neurons in phFTD may be able to provide a better functioning compensational mechanism than in bvFTD. The observation that the inferior parietal lobule showed both increased and decreased (depending on its subregion) functional connectivity in bvFTD in comparison with controls is in line with this view. It is plausible that the various subregions of the inferior parietal lobule are not affected to the same extent in bvFTD, resulting in decreased functional connectivity in the more affected subregion and in increased functional connectivity in the less affected subregion. Future studies consisting of a larger patient sample could shed further light on whether other resting-state networks show similar functional connectivity changes to the DMN. A network of particular interest would be the salience network, which is a frontal functional network known to be affected in bvFTD [13]. This would allow for further meaningful comparisons between phFTD and bvFTD.

Frontotemporal and parietal microstructural WM abnormalities were observed in both phFTD and bvFTD. In phFTD, FA was decreased (i.e. there was less directional diffusion) and RD and MD were increased (i.e. there was more diffusion, particularly perpendicular to the tract’s axis) in multiple WM tracts, including the cingulum, UF, IFOF, genu of the CC and SLF. Damage to these WM tracts has been linked to the various cognitive functions typically affected in bvFTD. Loss of behavioural control (e.g. disinhibition) has been related to diffusion changes in the UF, forceps minor and cingulum [32, 33]. Additionally, abnormalities in executive functioning, visuo-spatial attention, working memory and apathy have also been linked with diffusivity changes in the cingulum [34, 35]. Interestingly, RD changes in these tracts were less pronounced than FA changes, which may be explained by FA being a composite measure of both RD and AxD (i.e. diffusion along the tract’s main axis), and therefore more sensitive to subtle myelin and/or axonal changes, reflected by changes in RD [36] and AxD [37] respectively. In phFTD, AxD abnormalities were not observed, which may be explained by myelin damage only, without axonal injury. In bvFTD, WM changes were more pronounced and widespread, with lower FA and higher MD, RD and AxD than in phFTD. Hence, here we show an association in phFTD, similar to bvFTD, between symptomatology and damage to the frontotemporal and parietal WM tracts. Additionally, we show differences possibly reflecting neuropathological changes between phFTD and bvFTD, with phFTD suggesting myelin damage only and bvFTD showing more pronounced myelin and axonal damage.

Previous literature has shown a relationship between microstructural WM changes and functional connectivity changes [38–40] and proposes that microstructural WM predicts, or is reflected by, functional connectivity [39, 40]. For example, the medial prefrontal cortex and posterior cingulate cortex, core regions of the DMN, are connected through the cingulum [39]. Cingulum abnormalities such as observed in this study may have— to a certain extent—disconnected these regions, reducing the functional connectivity between the anterior and posterior DMN regions. In support of this idea, there were more pronounced anterior WM abnormalities in bvFTD that extended more posteriorly than in phFTD, and both frontal and parietal functional connectivity were seen to be correspondingly lower. A recent study by Weiler et al. (2014) [41] observed that higher RD in the cingulum and parahippocampal bundle (both connecting DMN regions) predicted reduced performance on measures related to DMN cognitive functions. We therefore hypothesise that more advanced abnormalities of WM tracts of the DMN may eventually lead to a functional decrease in the associated DMN areas and result in reduced cognitive functioning. As we did not aim to directly explore such a mechanism, our study design does not allow for any firm conclusions concerning this mechanism. A longitudinal design employing correlational analyses is needed to verify this hypothesis.

Overall, phFTD showed functional connectivity and subtle WM changes, whereas bvFTD showed fewer functional connectivity and more extensive WM changes. Lower overall cortical GM volume in phFTD patients was observed in this study using a quantitative method, but was not observed in the regular clinical diagnostic work-up. This suggests that GM volume loss is also present in phFTD patients, but at such a limited degree that it is not clinically detected. These findings are indicative of incipient degeneration in phFTD. In order to investigate phFTD without the interference of an alternative diagnosis we ruled out alternative psychiatric disorders, neuropsychological progression and presence of the C9orf72 mutation. The observed incipient brain changes in this well-defined population are in favour of the controversial notion that phFTD and bvFTD may belong to the same disease spectrum. PhFTD presents with behavioural, neuropsychological and, as shown here, also neurodegenerative changes that are all similar to those observed in bvFTD.

This study has some limitations. First, we were only able to investigate a small number of phFTD and bvFTD patients, limiting the statistical power. As a result, rs-fMRI effects, already expected to be subtle, were only detectable using a relatively lenient statistical threshold and could only be investigated in one functional network. The sample size is inherent to the rarity of the phFTD syndrome, together with the application of strict inclusion and exclusion criteria to avoid inadvertent inclusion of patients with bvFTD or alternative psychiatric disorders. Second, the bvFTD group was not fully matched for gender with the phFTD and control group. There is no conclusive evidence on gender differences in functional connectivity [42] or microstructural WM. Both higher and lower FA was measured in the cingulum and in the WM underlying the frontal cortex in men and in women compared with the opposite gender [43]. Given these findings it is not likely that the FA decreases observed in this study were driven by gender differences. Third, the bvFTD group and part of the control group were scanned on a different scanner, although of identical type and field strength, and with identical protocols. While a scanner effect cannot be excluded, both rs-fMRI and DTI have been shown to be highly reproducible in terms of DMN functional connectivity and TBSS respectively, even across different scanner platforms and vendors [44, 45]. Moreover, the fact that our findings in bvFTD are consistent with the previous literature suggests that the scanner effect is likely to be minimal.

In conclusion, our findings are in support of the hypothesis that phFTD and bvFTD may belong to the same disease spectrum. In phFTD, there are changes in functional connectivity and microstructural WM that are similar to those found in bvFTD. Advanced MRI techniques, such as rs-fMRI and DTI, are therefore potentially suited to improve the diagnosis of phFTD by identifying such incipient changes. Naturally, the hypothesis that phFTD and bvFTD may belong to the same disease spectrum would require confirmation with other diagnostic tools, such as histopathology. Also, further assessment in a longitudinal study to assess changes over time would be required, at which our future efforts are aimed. Our findings could provide a direction for further development of MR—or other—diagnostic tools.

## Electronic supplementary material

Below is the link to the electronic supplementary material.

ESM 1 (DOCX 42 kb)

## Abbreviations

phFTD: Phenocopy frontotemporal dementia
