# Supplementary material for: Functional connectivity and microstructural white matter changes in phenocopy frontotemporal dementia
Source: Eur Radiol. 2016 Jul 19;27(4):1352–60. doi: 10.1007/s00330-016-4490-4 (PMC5334426; doi:10.1007/s00330-016-4490-4)
Supplement: Supplementary file 1 — (DOCX 42 kb) [file 330_2016_4490_MOESM1_ESM.docx]

Supplement

**Supplement section 1**

Rs-fMRI analysis

Resting state data were analyzed using FMRIB Software library (FSL4.1.9, Oxford, UK)[1–3]. T1w images were first reoriented to ensure the same orientation as the standard template T1 images. The skull was stripped using the Brain Extraction Tool (BET)[4].

The Multivariate Exploratory Linear Optimized Decomposition into Independent Components (MELODIC) toolbox was used to preprocess the data and to perform independent component analysis (ICA). Preprocessing consisted of several steps. First the high pass filter was set to remove low-frequency drifts (cut-off 120s). Then MCFLIRT[5] motion correction was applied to correct for linear motion followed by spatial smoothing performed with a Gaussian kernel of 5 FWHM. During registration, functional data were first linearly registered to the corresponding T1w images (full search, 7 degrees of freedom (DOF)). This was followed by both linear and nonlinear registration to a standard template brain (full search, 12 DOF) with a warp resolution of 10mm and a resampling resolution of 4mm. Resting state networks were identified by performing a multi-session temporal concatenation ICA. Component output was limited to 30. Dual regression[6] was used to identify the group components in the functional data of each participant. The component representing the DMN was identified. The DMN was chosen because it is an anatomically clearly defined network and shows pronounced abnormalities in bvFTD patients [7,8]. Only one network was investigated because of the small sample size of the study and thus reduced statistical power. FSLcc was used to identify the component showing the highest correlation between a DMN template[9] and ICA output.

The Randomize[10] tool was used to assess between-group differences in DMN network connectivity. The design was constructed using the general linear model (GLM) toolbox. A one-way ANCOVA with three groups (phFTD≠bvFTD≠controls) was performed to assess which regions showed different connectivity between groups. Effects per group were investigated and six t-contrasts (phFTD>HC, HC>phFTD, bvFTD>HC, HC>bvFTD, phFTD>bvFTD, bvFTD>phFTD) were constructed to assess post-hoc between-group differences. Grey matter volume was added as covariate to account for grey matter atrophy. Threshold-free cluster enhancement (TFCE) [11] and nonparametric permutation testing with 5000 permutations was applied. Results were not family-wise error corrected for multiple comparisons on a voxel level. However, the f-test resulting from the one-way ANOVA and the group effects (phFTD, bvFTD, controls) were Bonferroni corrected for the number of contrasts, thus using an effective threshold of p<0.0125. Subsequent Bonferroni correction for the number of post-hoc t-tests was considered unnecessary as their results were assessed within the constraints of the omnibus f-test. This was done by creating common binary masks to identify post-hoc t-tests results within the omnibus f-test positive connectivity regions. Using the FSLmaths tool, first binary masks were created for the f-test and for every t-test. Second, the binary mask of each t-test was multiplied with binary mask of the f-test, resulting in common binary masks for each t-test.

All t-test common binary masks (p<0.05, not corrected for multiple comparisons), the f-test and the three group effects (p<0.05, Bonferroni corrected for multiple contrasts) were evaluated using the Cluster tool to extract cluster size with a cluster threshold of k≥20 voxels.

Results were visualised using FSLview. The Harvard-Oxford Cortical Structural Atlas and the Harvard-Oxford Subcortical Structural Atlas implemented in FSLView were used to anatomically identify the DMN regions.

DTI analysis

Data were corrected for motion, eddy currents and EPI distortions using ExploreDTI[12]. Further analyses were performed with FSL (5.0.2.2, Oxford, UK)[1–3]. BET[4] was used to create skull-stripped binary-masks. Using DTIFIT, as implemented in FMRIB’s Diffusion Toolbox (FDT)[13], the diffusion tensors were reconstructed. Then DTIFIT created subject images for fractional anisotropy (FA), mean diffusivity (MD), the first eigenvalue/axial diffusivity (AxD) and the second and third eigenvalues. FSLmaths was used to calculate subject radial diffusivity (RD) images by averaging the second and third eigenvalue images.

Registration of diffusivity measure images was executed by Tract-Based Spatial Statistics (TBSS) [14]. First, subject FA image outliers from DTIFIT were excluded by removing brain-end artefacts and end slices. Second, non-linear registration of all subject FA images to the FMRIB58 FA template was performed. Third, all subject FA images were normalized to the Montreal Neurological Institute (MNI) template. Then the mean of all normalized subject FA images was taken to create a mean FA skeleton. Fifth, the mean FA skeleton was binarised. Then each subject’s FA image was projected onto this binary FA skeleton. TBSS was repeated for AxD, MD and RD images. FA nonlinear registration parameters were applied to subject AxD, MD and RD images creating nonlinearly registered images for each of them. For each measure all subject images were projected onto the FA skeleton, creating a new skeleton for each diffusivity measure.

Randomize[10] was run to test for between-group differences in all described diffusivity measures. TFCE[11] nonparametric permutation testing with 5000 permutations was applied. Results were family-wise error (FWE) corrected for multiple comparisons.

WM microstructure differences between phFTD, bvFTD and controls were assessed using the same design defined for the rs-fMRI analysis, but now only the f-test and subsequent post-hoc t-tests were investigated. As group effects were not investigated, additional Bonferroni correction for the number of contrast was unnecessary. Again, common binary masks were created to assess t-test results within the constraints of the omnibus f-test results by multiplying binary masks of each t-test with the binary mask of the f-test. All t-test common binary masks (p<0.05, FWE corrected for multiple comparisons) were evaluated using Cluster to extract cluster size with a cluster threshold of k≥20.

Results were visualised using FSLView. The JHU White-Matter Tractography Atlas and the JHU ICBM-DTI-81 White-Matter labels implemented in FSLView were used to anatomically identify the white matter regions.

References

1 Jenkinson M, Beckmann CF, Behrens TEJ, *et al.* FSL. *Neuroimage* 2012;**62**:782–90.

2 Woolrich MW, Jbabdi S, Patenaude B, *et al.* Bayesian analysis of neuroimaging data in FSL. *Neuroimage* 2009;**45**:S173–86.

3 Smith SM, Jenkinson M, Woolrich MW, *et al.* Advances in functional and structural MR image analysis and implementation as FSL. *Neuroimage* 2004;**23 Suppl 1**:S208–19.

4 Smith SM. Fast robust automated brain extraction. *Hum Brain Mapp* 2002;**17**:143–55.

5 Jenkinson M, Bannister P, Brady M, *et al.* Improved optimization for the robust and accurate linear registration and motion correction of brain images. *Neuroimage* 2002;**17**:825–41.

6 Filippini N, MacIntosh BJ, Hough MG, *et al.* Distinct patterns of brain activity in young carriers of the APOE-epsilon4 allele. *Proc Natl Acad Sci U S A* 2009;**106**:7209–14.

7 Zhou J, Greicius MD, Gennatas ED, *et al.* Divergent network connectivity changes in behavioural variant frontotemporal dementia and Alzheimer’s disease. *Brain* 2010;**133**:1352–67.

8 Buckner RL, Andrews-Hanna JR, Schacter DL. The brain’s default network: anatomy, function, and relevance to disease. *Ann N Y Acad Sci* 2008;**1124**:1–38.

9 Beckmann CF, DeLuca M, Devlin JT, *et al.* Investigations into resting-state connectivity using independent component analysis. *Philos Trans R Soc L B Biol Sci* 2005;**360**:1001–13.

10 Winkler AM, Ridgway GR, Webster MA, *et al.* Permutation inference for the general linear model. *Neuroimage* 2014;**92**:381–97.

11 Smith SM, Nichols TE. Threshold-free cluster enhancement: addressing problems of smoothing, threshold dependence and localisation in cluster inference. *Neuroimage* 2009;**44**:83–98.

12 Leemans A, Jeurissen B, Sijbers J, *et al.* ExploreDTI: a graphical toolbox for processing, analyzing, and visualizing diffusion MR data. In: *Proceedings 17th Scientific Meeting, International Society for Magnetic Resonance in Medicine*. 2009. 3537.http://www.mendeley.com/research/exploredti-a-graphical-toolbox-for-processing-analyzing-and-visualizing-diffusion-mr-data/\nhttp://www.exploredti.com/ref/ExploreDTI_ISMRM_2009.pdf

13 Behrens TEJ, Woolrich MW, Jenkinson M, *et al.* Characterization and propagation of uncertainty in diffusion-weighted MR imaging. *Magn Reson Med* 2003;**50**:1077–88.

14 Smith SM, Jenkinson M, Johansen-Berg H, *et al.* Tract-based spatial statistics: voxelwise analysis of multi-subject diffusion data. *Neuroimage* 2006;**31**:1487–505.

**Supplement section 2 – Participant exclusion after inclusion**

Nine phFTD patients (all male), 12 bvFTD patients (seven male) and 20 healthy controls (all male) were included in the study. Two phFTD patients were excluded from the analysis: one refused neuropsychological testing and one showed disease progression on neuropsychological testing. One phFTD patient showed a cortical infarct in the right parietal lobe on the structural MRI scan, but reported no residual clinical symptoms and his neuropsychological profile was rated as normal; he was therefore retained in the analysis. Three controls were excluded from the analysis: one due to a below average score on a neuropsychological domain typically affected in FTD; the second due to an incidental scanner artefact observed in the MR images; the third because of missing DTI data. Three bvFTD patients had missing rs-fMRI data.

Supplement Table 1A. Group effects (p<0.05, not corrected for multiple comparisons, but Bonferroni corrected (p<0.05) for multiple contrasts; k≥20) of DMN connectivity in phFTD, bvFTD and controls.

DMN = default mode network, phFTD = phenocopy frontotemporal dementia, bvFTD = behavioural variant frontotemporal dementia, HC = healthy controls, L = left, R = right.

| **Group effect** | **Number of DMN clusters** | **Total number of voxels** | **Number of voxels within largest DMN cluster** | **Anatomical regions within largest DMN cluster** |
| --- | --- | --- | --- | --- |
| phFTD | 2 | 7,852 | 7,827 | Medial prefrontal cortex L, R  Lateral temporal cortex L, R  Inferior parietal lobule L, R  Precuneus/posterior cingulate cortex L, R |
| bvFTD | 2 | 4,848 | 4,458 | Lateral temporal cortex L, R  Inferior parietal lobule L, R  Precuneus/posterior cingulate cortex L, R |
| HC | 5 | 1,635 | 1,015 | Inferior parietal lobule R  Precuneus/posterior cingulate cortex L, R |

Supplement Table 1B. Post-hoc two sample t-tests (p<0.05, not corrected for multiple comparisons, but within the constraints of the omnibus f-test (p<0.05, Bonferroni corrected for multiple contrasts); k≥20) of group differences in DMN connectivity between phFTD, bvFTD and controls.
DMN = default mode network, phFTD = phenocopy frontotemporal dementia, bvFTD = behavioural variant frontotemporal dementia, HC = healthy controls, L = left, R = right.

| **T-stat** | **Number of DMN clusters** | **Total number of voxels** | **Number of voxels within largest DMN cluster** | **Anatomical regions within largest DMN cluster** |
| --- | --- | --- | --- | --- |
| phFTD>HC | 7 | 635 | 162 | Lateral temporal cortex R  Inferior parietal lobule R |
| bvFTD>HC | 3 | 104 | 43 | Lateral temporal cortex R  Inferior parietal lobule R |
| HC>bvFTD | 1 | 44 | 44 | Inferior parietal lobule R |
| phFTD>bvFTD | 7 | 852 | 374 | Lateral temporal cortex L  Subcortical and cerebellar regions L |

Supplement Table 2A. Post-hoc two sample t-tests (p_corrected_<0.05; k≥20) of group differences in FA between phFTD, bvFTD and controls.
FA = fractional anisotropy, phFTD = phenocopy frontotemporal dementia, bvFTD = behavioural variant frontotemporal dementia, HC = healthy controls, L = left, R = right.

| **T-stat** | **Number of clusters** | **Total number of voxels** | **Number of voxels within largest cluster** | **Anatomical regions within largest cluster** |
| --- | --- | --- | --- | --- |
| phFTD<HC | 28 | 14,084 | 4,644 | Forceps minor  Genu of the corpus callosum L, R  Body of the corpus callosum L, R  Inferior fronto-occipital fasciculus L, R  Anterior thalamic radiation L, R  Cingulum L, R  Uncinate fasciculus L, R |
| bvFTD<HC | 12 | 31,220 | 29,598 | Forceps minor  Forceps major  Genu of the corpus callosum L, R  Body of the corpus callosum L, R  Splenium of the corpus callosum L, R  Inferior fronto-occipital fasciculus L, R  Anterior thalamic radiation L, R  Cingulum L, R  Uncinate fasciculus L, R  Inferior longitudinal fasciculus L, R  Superior longitudinal fasciculus L, R |
| bvFTD<phFTD | 4 | 8,025 | 7,777 | Forceps minor  Genu of the corpus callosum L, R  Body of the corpus callosum L, R  Inferior fronto-occipital fasciculus L, R  Anterior thalamic radiation L, R  Cingulum L, R  Uncinate fasciculus L, R |

Supplement Table 2B. Post-hoc two sample t-tests (p_corrected_<0.05; k≥20) of group differences in MD between phFTD, bvFTD and controls.
MD = mean diffusivity, phFTD = phenocopy frontotemporal dementia, bvFTD = behavioural variant frontotemporal dementia, HC = healthy controls, L = left, R = right.

| **T-stat** | **Number of clusters** | **Total number of voxels** | **Number of voxels within largest cluster** | **Anatomical regions within largest cluster** |
| --- | --- | --- | --- | --- |
| phFTD>HC | 5 | 1,686 | 883 | Anterior thalamic radiation L, R  Inferio fronto-occipital fasciculus L, R  Superior longitudinal fasciculus L, R |
| bvFTD>HC | 14 | 39,036 | 37,066 | Forceps minor  Forceps major  Genu of the corpus callosum L, R  Body of the corpus callosum L, R  Splenium of the corpus callosum L, R  Inferior fronto-occipital fasciculus L, R  Anterior thalamic radiation L, R  Cingulum L, R  Uncinate fasciculus L, R  Inferior longitudinal fasciculus L, R  Superior longitudinal fasciculus L, R |
| bvFTD>phFTD | 4 | 19,769 | 19,586 | Forceps minor  Genu of the corpus callosum L, R  Body of the corpus callosum L, R  Inferior fronto-occipital fasciculus L, R  Anterior thalamic radiation L, R  Cingulum L, R  Uncinate fasciculus L, R  Inferior longitudinal fasciculus L  Superior longitudinal fasciculus L, R |

Supplement Table 2C. Post-hoc two sample t-tests (p_corrected_<0.05; k≥20) of group differences in RD between phFTD, bvFTD and controls.
RD = radial diffusivity, phFTD = phenocopy frontotemporal dementia, bvFTD = behavioural variant frontotemporal dementia, HC = healthy controls, L = left, R = right.

| **T-stat** | **Number of clusters** | **Total number of voxels** | **Number of voxels within largest cluster** | **Anatomical regions within largest cluster** |
| --- | --- | --- | --- | --- |
| phFTD>HC | 9 | 7,963 | 6,137 | Forceps minor  Forceps major  Inferior fronto-occipital fasciculus R  Anterior thalamic radiation R  Cingulum R  Uncinate fasciculus R  Inferior longitudinal fasciculus R  Superior longitudinal fasciculus R |
| bvFTD>HC | 16 | 46,485 | 45,111 | Forceps minor  Forceps major  Genu of the corpus callosum L, R  Body of the corpus callosum L, R  Splenium of the corpus callosum L, R  Inferior fronto-occipital fasciculus L, R  Anterior thalamic radiation L, R  Cingulum L, R  Uncinate fasciculus L, R  Inferior longitudinal fasciculus L, R  Superior longitudinal fasciculus L, R |
| bvFTD>phFTD | 6 | 22,230 | 21,946 | Forceps minor  Genu of the corpus callosum L, R  Body of the corpus callosum L, R  Inferior fronto-occipital fasciculus L, R  Anterior thalamic radiation L, R  Cingulum L, R  Uncinate fasciculus L, R  Inferior longitudinal fasciculus L, R  Superior longitudinal fasciculus L, R |

Supplement Table 2D. Post-hoc two sample t-tests (p_corrected_<0.05; k≥20) of group differences in AxD between phFTD, bvFTD and controls.
AxD = axial diffusivity, phFTD = phenocopy frontotemporal dementia, bvFTD = behavioural variant frontotemporal dementia, HC = healthy controls, L = left, R = right.

| **T-stat** | **Number of clusters** | **Total number of voxels** | **Number of voxels within largest cluster** | **Anatomical regions within largest cluster** |
| --- | --- | --- | --- | --- |
| phFTD>HC | 0 | n/a | n/a | n/a |
| bvFTD>HC | 3 | 18,269 | 17,552 | Forceps minor  Genu of the corpus callosum L, R  Body of the corpus callosum L, R  Inferior fronto-occipital fasciculus L, R  Anterior thalamic radiation L, R  Cingulum L  Uncinate fasciculus L, R  Superior longitudinal fasciculus L, R |
| bvFTD>phFTD | 8 | 11,202 | 10,396 | Forceps minor  Genu of the corpus callosum L, R  Body of the corpus callosum L, R  Inferior fronto-occipital fasciculus L, R  Anterior thalamic radiation L, R  Uncinate fasciculus L, R  Superior longitudinal fasciculus L, R |
